# Exercise training improves exercise capacity and quality of life in heart failure with preserved ejection fraction: a systematic review and meta-analysis of randomized controlled trials

**DOI:** 10.1093/ehjopen/oeae033

**Published:** 2024-06-26

**Authors:** Ranu Baral, Jamie Sin Ying Ho, Ayesha Nur Soroya, Melissa Hanger, Rosemary Elizabeth Clarke, Sara Fatima Memon, Hannah Glatzel, Mahmood Ahmad, Rui Providencia, Jonathan James Hyett Bray, Fabrizio D’Ascenzo

**Affiliations:** Kings College Hospital NHS Trust, London, Denmark Hill, London SE5 9RS, UK; Royal Free London NHS Foundation Trust, Pond St, London NW3 2QG, UK; University College London, Gower St, London WC1E 6BT, UK; University College London, Gower St, London WC1E 6BT, UK; University College London, Gower St, London WC1E 6BT, UK; University College London, Gower St, London WC1E 6BT, UK; Stoke Mandeville Hospital, Mandeville Rd, Aylesbury HP21 8AL, UK; Royal Free London NHS Foundation Trust, Pond St, London NW3 2QG, UK; University College London, Gower St, London WC1E 6BT, UK; Barts Heart Centre, Barts Health NHS Trust, W Smithfield, London EC1A 7BE, UK; Institute of Life Sciences-2, Swansea Bay University Health Board and Swansea University Medical School, Swansea University, Sketty, Swansea SA2 8QA, UK; Division of Cardiology, Department of Medical Science, University of Turin, Via Verdi 8, 10124 Torino, P.I. 02099550010, Italy

**Keywords:** Exercise training, Heart failure, Meta-analysis, Heart failure with preserved ejection fraction, HFpEF

## Abstract

**Aims:**

Heart failure with preserved ejection fraction (HFpEF) is associated with high morbidity and mortality, and there are limited proven therapeutic strategies. Exercise has been shown to be beneficial in several studies. We aimed to evaluate the efficacy of exercise on functional, physiological, and quality-of-life measures.

**Methods and results:**

A comprehensive search of Medline and Embase was performed. Randomized controlled trials (RCTs) of adult HFpEF patients with data on exercise intervention were included. Using meta-analysis, we produced pooled mean difference (MD) estimates with 95% confidence intervals (CIs) with Review Manager (RevMan) software for the peak oxygen uptake (VO_2_), Minnesota living with heart failure (MLWHF) and, other diastolic dysfunction scores. A total of 14 studies on 629 HFpEF patients were included (63.2% female) with a mean age of 68.1 years. Exercise was associated with a significant improvement in the peak VO_2_ (MD 1.96 mL/kg/min, 95% CI 1.25–2.68; *P* < 0.00001) and MLWHF score (MD −12.06, 95% CI −17.11 to −7.01; *P* < 0.00001) in HFpEF. Subgroup analysis showed a small but significant improvement in peak VO_2_ with high-intensity interval training (HIIT) vs. medium-intensity continuous exercise (MCT; MD 1.25 mL/kg/min, 95% CI 0.41–2.08, *P* = 0.003).

**Conclusion:**

Exercise increases the exercise capacity and quality of life in HFpEF patients, and high-intensity exercise is associated with a small but statistically significant improvement in exercise capacity than moderate intensity. Further studies with larger participant populations and longer follow-up are needed to confirm these findings and elucidate potential differences between high- and medium-intensity exercise.

## Graphical Abstract

**Graphical Abstract:**
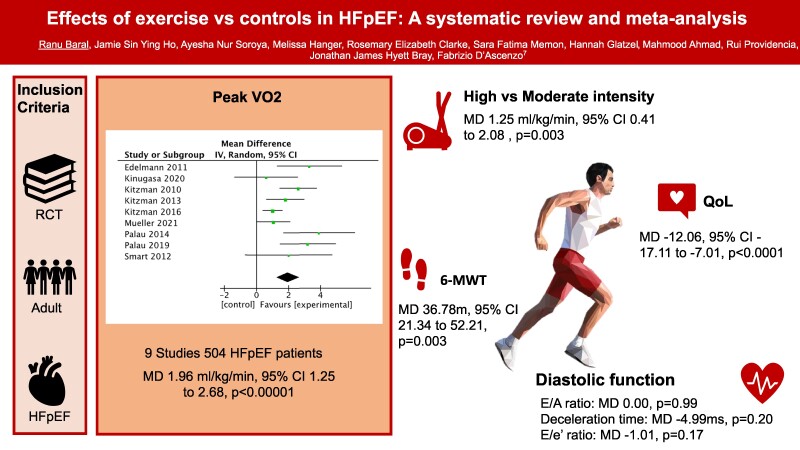

## Introduction

Heart failure is recognized as an increasing public health burden, affecting ∼920 000 people in the UK and costing $108 billion/year worldwide.^1^ Heart failure with preserved ejection fraction (HFpEF) represents >50% of heart failure diagnosis in patients older than 65 years of age.^2^ It is increasing in prevalence^3^ and is also associated with high morbidity and mortality, increased re-hospitalization rates,^4^ and a worse quality of life.^5^ There has been an extensive search for novel therapeutics to improve symptom control, and exercise training is shown to have promising results.^6,7^

While the current European Society of Cardiology guidelines^8^ encourage exercise in HFpEF to improve exercise capacity, evidence for specific types or duration of exercise regimens is scarce. Several recent studies have found conflicting evidence on the relative benefit of high-intensity interval training (HIIT) and moderate-continuous training (MCT).^9,10^ High-intensity interval training, involving short work periods at higher intensity and high submaximal load, has been shown to be superior to continuous exercise in patients with coronary artery disease and left ventricular systolic dysfunction.^10,11^ Several small trials in HFpEF found that HIIT was superior to MCT,^12^ but the recent moderate-sized OptimEx-Clin RCT by Mueller *et al*.^9^ did not confirm these findings. Furthermore, while previous RCTs generally explored exercise regimens of several months, several RCTs of longer duration, up to 1 year, have been recently published.^9,13^ It is, therefore, important to further explore the optimal exercise regimen in this growing population of HFpEF patients with few treatment options, and an updated meta-analysis is warranted.

We aimed to systematically review the effect of exercise training on the cardiorespiratory function, quality of life, and diastolic function of patients with HFpEF. We also investigated the outcomes of exercise regimens of different intensities and duration in patients with HFpEF.

## Methods

### Search strategy

The systematic review was conducted and reported in accordance with the Preferred Reporting Items for Systematic Reviews and Meta-Analyses (PRISMA) guidelines. A comprehensive literature search of Medline and Embase was conducted from inception to December 2022 using MeSH terms and keywords (see Supplementary material online, *Figure S1*). Reference lists of included studies and review articles were hand-searched for additional studies. Randomized controlled trials with adult HFpEF patients with data on exercise intervention, including aerobic or endurance exercise, resistance training, HIIT, inspiratory muscle training (IMT), or a combination of the above were included. Studies performed on patients with HFrEF, non-adult patients, and pre-clinical studies were excluded. Studies published in languages other than English were excluded.

### Study selection and data extraction

All studies identified in our search were primarily screened using the titles and the abstracts. Each article was independently screened by at least two authors (R.B., S.F.M., A.N.S., R.E.C., M.H.) and any discrepancies were resolved by discussion and involvement of a senior author (M.A.) if necessary. Any article identified as having a potential of fulfilling our inclusion criteria underwent full-text evaluation. Among trials studying the same population with multiple publications, the trial with the maximum number of patients was included. For the included studies, relevant information such as the type of the study, characteristics of the participants, and number of patients in each group was extracted independently by two authors (S.F.M., A.N.S., R.E.C., M.H.) onto a standardized form.

### Outcome measures

The primary outcomes of the study were change in the peak oxygen uptake (peak VO_2_) in mL/kg/min, 6 min walk test (6-MWT), Minnesota living with heart failure (MLWHF) score, and markers of diastolic function (changes in *E*/*A* ratio, *E*/*e*′ ratio, and early deceleration time).

### Risk of bias assessment and publication bias

The assessment of the quality of each included studies was performed independently by two authors. The Cochrane Collaboration tool for assessing the risk of bias was used to assess the quality of included randomized studies^14^ and funnel plot asymmetry was used to assess publication bias when ≥10 studies were available.

### Statistical analysis

The data were analysed using random effects analysis in Review Manager (RevMan) software (Version 5.3. Copenhagen: The Nordic Cochrane Centre, The Cochrane Collaboration, 2014). Open Meta[Analyst] Software version 10.12 (developed by the Centre for Evidence Synthesis, Brown University, School of Public Health, Rhode Island State, USA) was used for meta-regression.^15^ Mean differences (MDs) with corresponding 95% confidence intervals (95% CIs) were calculated for continuous outcomes. In studies with missing standard deviations (SDs) for change from baseline data, SD was calculated using the correlation coefficient formula.^16^

A subgroup analysis of studies evaluating HIIT vs. MCT, endurance training vs. non-endurance training, length, and number of sessions in exercise regimen was performed. As previous study^17^ has shown the achievement of more METs (metabolic equivalent of tasks), higher peak HR, and lower resting HR in a 12-week programme compared with 6 weeks and British Heart Foundation (BHF) recommends 10–12 weeks of cardiac rehabilitation,^18^ 12 weeks were taken as the cut-off for the subgroup. For the number of sessions, a median value was calculated to divide the subgroups.

Statistical heterogeneity was evaluated by calculating *I*^2^ statistics, with the prespecified cut-offs of low heterogeneity defined as *I*^2^ value of <25%, moderate heterogeneity was 25–75%, and high heterogeneity was >75%.^19^ The statistical significance was defined as *P* < 0.05.^20^

## Results

**Figure 1:**
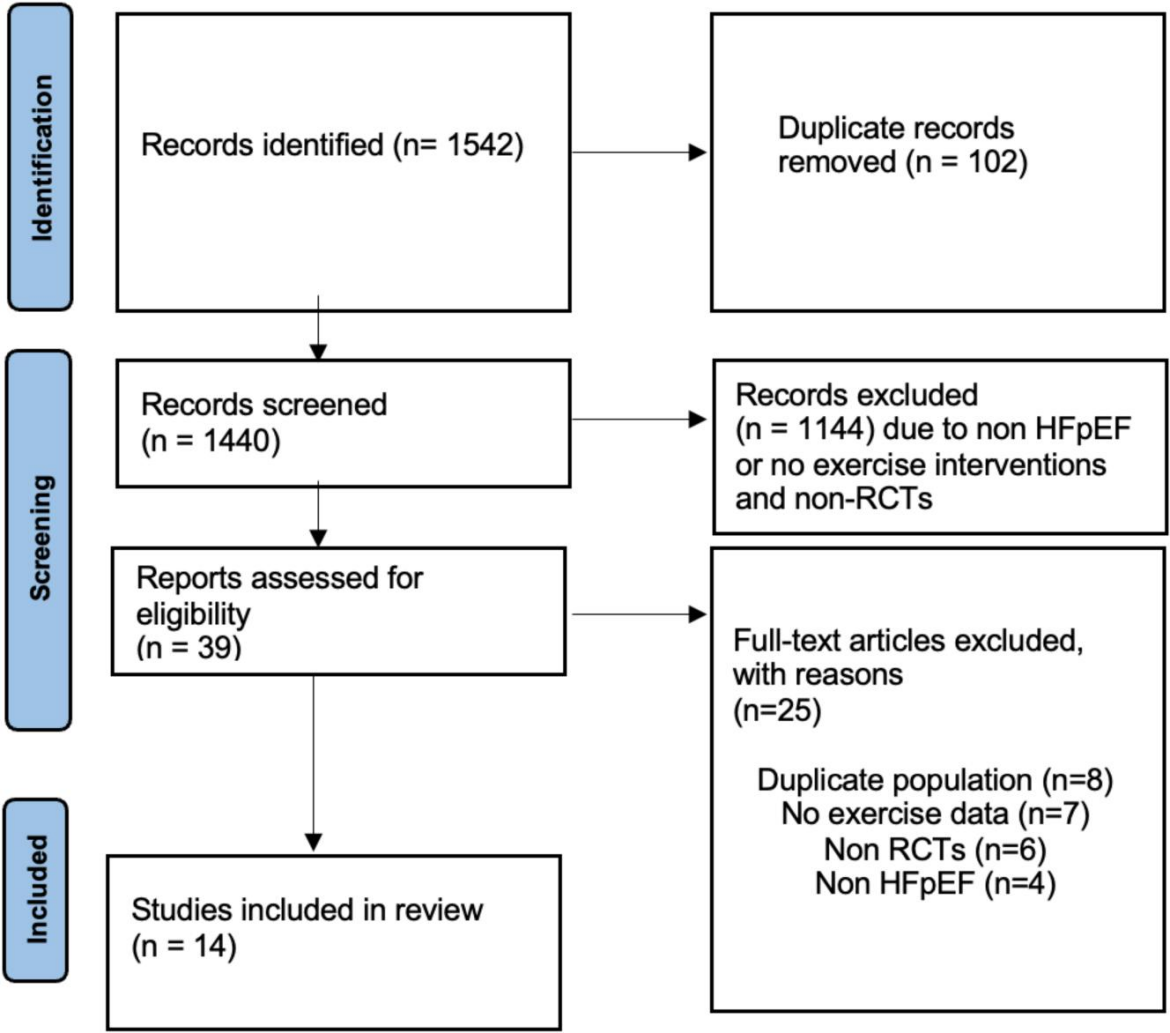
Preferred reporting items for systematic reviews and meta-analyses (PRISMA) flow diagram of the trial selection process.

Our initial comprehensive search of the literature sources identified 1542 entries; of which, 14 articles^6,7,9,12,21–30^ were included in the final analysis (*Figure 1*). There were 629 participants in total, with a mean age of 68.1 years and 63.2% were female. All studies, except three,^6,7,21^ used EF >50% as the cut-off for HFpEF. Baseline characteristics of the participants are given in *Table 1*. The range of follow-up was 4–52 weeks. Ten studies used endurance exercise training in the intervention group vs. usual care in the control group, three studies^26–28^ used inspiratory muscle training, and one study^29^ used function electrical stimulation using direct electrical current at 25 Hz as a method of exercise training (*Table 2*).

**Table 1:** Baseline characteristics of the studies included in the meta-analysis

Author, year	*n* (control/exercise)	♀	White, %	Mean age, year	Mean BMI, kg/m^2^	HTN, %	DM, %	AF, %	Exercise Type	Total sessions	Duration, weeks
		%									
Gary *et al* 2004^6^	13/15	100	59	68 ± 11	34 ± 7	88	31	NR	Walking	36	12
Kitzman *et al*. 2010^23^	27/26	75	70	69 ± 5	31 ± 6	68	17	NR	Walking, cycling	48	16
Edelmann *et al* 2011^30^	20/44	56	NR	65 ± 7	31 ± 5	86	14	NR	Cycling, resistance training	32	12
Alves *et al* 2012^21^	11/20	29	NR	63 ± 10	28 ± 5	68	35	3	Walking, cycling	72	24
Smart *et al* 2012^7^	13/12	48	NR	64 ± 6	32 ± 6	16	16	NR	Cycling	48	16
Karavidas *et al* 2013^29^	15/15	60	NR	69.0 ± 8.1	NR	100	47	40	Functional electrical stimulation	30	6
Kitzman *et al* 2013^24^	31/32	76	68	70 ± 7	32 ± 7	89	24	NR	Walking, cycling	48	16
Palau *et al* 2014^26^	12/14	50	NR	NR	NR	96	54	35	IMT	168	12
Angadi *et al* 2015^22^	6/9	20	NR	60 ± 6	30 ± 5	NR	27		Walking	12	4
Kitzman *et al* 2016^25^	25/26	80	61	67 ± 5	40 ± 7	96	37	2	Walking	60	20
Palau *et al* 2019^27^	13/15	61	NR	75 ± 9	32 ± 5	96	36	54	IMT	168	12
Kinugasa *et al* 2020^28^	12/8	15	NR	76 ± 10	NR	NR	NR	NR	IMT	168	24
Silveria 2020	9/10	63	NR	60 ± 9	33 ± 5	100	58	11	Walking	36	12
Mueller *et al* 2021^9^	60/116	69	NR	70 ± 7	30 ± 6	86	25	42	Cycling	156	52

DM, diabetes mellitus; AF, Atrial fibrillation; HTN, hypertension; IMT, inspiratory muscle training; NR, not reported.

**Table 2:** Types of exercise

Author, year	Exercise type
	**Endurance training**
Gary *et al* 2004^6^	Walking
Kitzman *et al*. 2010^23^	Walking, cycling
	Supervised aerobic exercise training, with exercise intensity increased to 60–70% of heart rate reserve
Edelmann *et al* 2011^30^	Cycling, resistance training
	Endurance and resistance training, with a target heart rate of 50–60% of peak VO2
Alves *et al* 2012^21^	Walking, cycling
	Interval training
Smart *et al* 2012^7^	Cycling
	Supervised, outpatient, cycle ergometer exercise training at 60 rpm, with initial intensity of 60–70% peak oxygen consumption and uptitrated by 2–5 Watts per week
Kitzman *et al* 2013^24^	Walking, cycling
	Endurance exercise training, with initially at 40–50% of heart rate, reserve and intensity increased gradually until 70% heart rate reserve
Angadi *et al* 2015^22^	Walking
	Treadmill training, with maximum 85–90% peak heart rate for HIIT
	Treadmill training, with maximum 70% peak heart rate for MCT
Kitzman *et al* 2016^25^	Walking exercise, and intensity level was progressed as tolerated and based primarily on heart rate reserve
Donelli da Silveria et al 2020^12^	Walking
	with 80–90% of peak VO_2_ and 85–95% of peak heart rate for HIIT
	50–60% of peak VO_2_ and 60–70% of peak heart rate for MCT
Mueller *et al* 2021^9^	Cycling
	with maximum 80–90% of heart rate reserve for HIIT
	with maximum 35–50% of heart rate reserve for MCT
	**Functional electrical stimulation**
Karavidas *et al* 2013^29^	Eight adhesive electrodes were positioned on the skin over quadriceps and gastrocnemius muscle of both legs. A direct electrical current at 25 Hz for 5 s followed by a 5 s rest. The patients were trained for 30 min a day, 5 days per week for a total of 6 weeks.
	**Inspiratory muscle training**
Palau *et al* 2014^26^	IMT
	Inspiratory muscle training twice a day (20 min per session) for 12 weeks, with breathing at a resistance equal to 25–30% and modified each session according to the 25–30% of inspiratory muscle training measured
Palau *et al* 2019^27^	IMT
	Inspiratory muscle training twice daily (20 min each session) for 12 weeks, resistance equal to 25%– 30% of their maximal inspiratory pressure for 1 week and was modified each session to 25–30% of their measured maximal inspiratory pressure
Kinugasa *et al* 2020^28^	Inspiratory muscle training once daily for 20 min for 24 weeks, resistance equal to 30% of their maximum inspiratory muscle pressure.

### Risk of bias assessment and publication bias

The Cochrane risk of bias assessing the quality of included studies is demonstrated in Supplementary material online, *Table S1*. All studies had at least two or more domains with unclear or high risk of bias. Publication bias was assessed using funnel plot asymmetry which was suggestive of small study bias (see Supplementary material online, *Figure S2*).

### Exercise capacity

**Figure 2:**
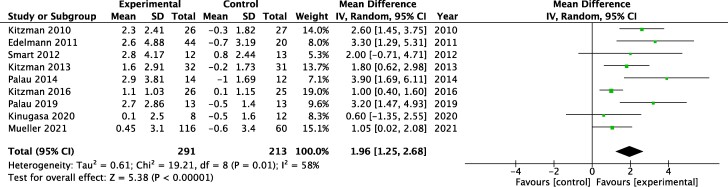
Forest plot showing the effect of exercise training on peak oxygen uptake (VO_2_) among participants with heart failure and preserved ejection fraction. CI, confidence interval; MD, mean difference.

Nine studies reported data on exercise capacity at baseline and after a period of exercise training, involving 504 HFpEF patients. Exercise was associated with a significant improvement in the peak VO_2_ (MD 1.96 mL/kg/min, 95% CI 1.25–2.68, *P* < 0.00001; *Figure 2*), with moderate heterogeneity (*I*^2^ = 58%). We also analysed 6-MWT to assess exercise capacity. There was a significant improvement in 6-MWT in the intervention group compared with control (MD 36.78 m, 95% CI 21.34–52.21, *P* < 0.00001; Supplementary material online, *Figure S3*).

**Figure 3:**
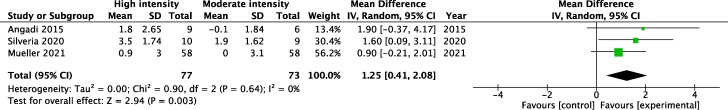
Subgroup analysis of studies investigating high- vs. moderate-intensity exercise. Forest plot showing the effect of exercise training on peak oxygen uptake (VO_2_) among participants with heart failure and preserved ejection fraction. CI, confidence interval; MD, mean difference.

Three studies^9,12,22^ on 150 patients compared the effect of HIIT vs. MCT in HFpEF. The exercise regime for these studies is outlined in *Table 1*. Overall, MCT was progressed to 60–70% of the peak heart rate (PHR) whilst HIIT was progressed to 80–95% of PHR. The PHR was considerably lower at 35–50% in the moderate subgroup in the Mueller study. Meta-analysis showed a significant improvement in the peak VO_2_ with HIIT compared with MCT (MD 1.25 mL/kg/min, 95% CI 0.41–2.08, *P* = 0.003, *Figure 3*).

### Quality of life

**Figure 4:**
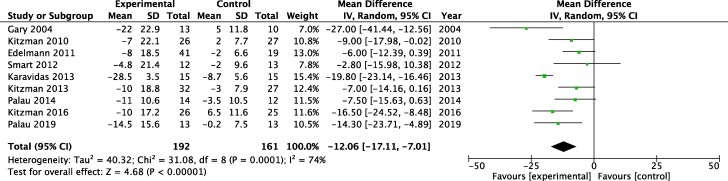
Effect of exercise training on quality of life. Forest plot showing the effect of exercise training quality of life, estimated using the Minnesota living with heart failure (MLWHF) score, among participants with heart failure and preserved ejection fraction. CI, confidence interval; MD, mean difference.

Nine studies reported an effect of exercise on quality of life determined using the MLWHF questionnaire. Apart from one study,^21^ all studies showed >5-point reduction MLWHF score, which has been denoted as a clinically meaningful improvement. Overall, there was a statistically significant improvement in the MLWHF score among exercise training group compared with controls (MD −12.06, 95% CI −17.11 to −7.01, *P* < 0.0001; *Figure 4*).

### Diastolic function

**Figure 5:**
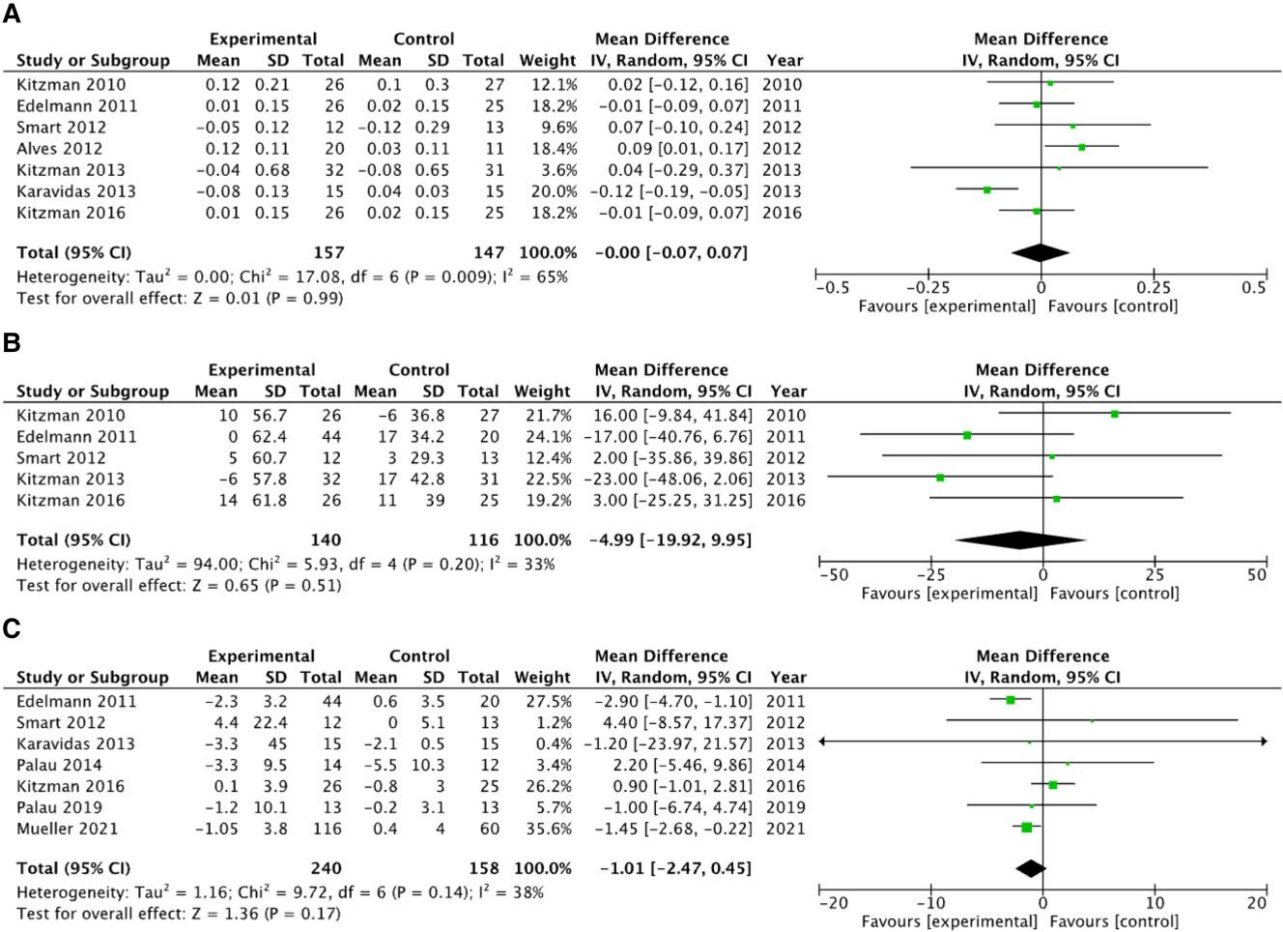
Effect of exercise training on diastolic function. Forest plot showing the effect of exercise training on the *E*/*A* ratio (*A*), deceleration time (*B*) on the *E*/*e*′ ratio (*C*) among participants with heart failure and preserved ejection fraction. CI, confidence interval; MD, mean difference.

Overall, in pooled analyses, exercise had no significant effect on diastolic function in HFpEF as measured by the *E*/*A* ratio (MD 0.00, 95% CI −0.07 to 0.07, *P* = 0.99; *Figure 5A*), deceleration time (MD −4.99 ms, 95% CI −19.92 to 9.95, *P* = 0.20; *Figure 5B*), and *E*/*e*′ ratio (MD −1.01, 95% CI −2.47 to 0.45, *P* = 0.17; *Figure 5C*).

### Subgroup analysis and meta-regression

To investigate the source of heterogeneity of the primary outcome analysis, we performed subgroup analysis and meta-regressions. Comparing endurance vs. non-endurance-based regimes, there was a trend for an increase in peak VO_2_ with non-endurance regimes (MD 1.59 mL/kg/min, 95% CI 1.19–1.98, *P* = 0.08, Supplementary material online, *Figure S4*). Additionally, our sub-analyses showed that both short (≤12 weeks) and longer programmes (≥16 weeks) improve peak VO_2_ (MD 3.41 vs. 1.43 mL/kg/min, *P* = 0.002, Supplementary material online, *Figure S5*) with the high heterogeneity (*I*^2^ = 89%) not allowing any clear inferences from comparison of subgroup treatment effects. The sub-analyses for the number of sessions showed benefit for programmes with ≤48 sessions and ≥60 sessions, with no significant subgroup differences observed when comparing the two groups (MD 2.34 vs. 1.7 mL/kg/min, *P* = 0.31, Supplementary material online, *Figure S6*). This was confirmed with meta-regression analysis which failed to generate significant interaction effects (co-efficient −0.053, *P* = 0.06; Supplementary material online, *Figure S7A* for duration, co-efficient 0.001, *P* = 0.93; Supplementary material online, *Figure S7B* for the number of sessions). Furthermore, sensitivity analysis without Karavidas *et al*.^29^ which applied functional electrical stimulation to assess the effect on the increase in MLWHF questionnaire showed no significant differences (see Supplementary material online, *Figure S8*).

## Discussion

Our meta-analysis of RCTs shows that training improves exercise capacity and quality of life in patients with HFpEF.^31,32^ Our analysis shows a benefit of exercise training even after a short number of sessions or weeks of training. Even though we could not observe a dose–response effect, study heterogeneity and the small number of included studies do not allow any sound inferences on this respect. High-intensity interval training led to a small but significant improvement in peak VO_2_ when compared with medium-intensity continuous exercise.

Previous meta-analyses.^33,34^ Guo *et al*.^34^ analysed 16 studies to demonstrate the effects of exercise in HFpEF, but the meta-analysis included non-RCTs and mixed studies with the same population. This also extends to similar recent meta-analysis.^32,35^ In this study, however, we used stringent criteria to exclude studies with the same population increasing result accuracy and included recently published newer studies. Secondly, diastolic function mainly signified by *E*/*A*, deceleration time, and *E*/*E*′ remained unchanged with exercise training.

The previous meta-analyses by Pandey *et al*.^33^ and Boulmpou *et al.*^32^ both showed that exercise training improved the peak exercise VO_2_ across four and eight studies, respectively.^31,32^ In our meta-analysis of 11 RCTs, exercise training was associated with a 1.96 mL/kg/min (95% CI 1.25–2.68). However, the increase in peak VO_2_ did not reach the prespecified clinically significant increase of 2.5 mL/kg/min set out in the OptimEx-Clin trial.^9^ Interestingly, subgroup analysis of studies comparing inspiratory training vs. aerobic (2.53 vs. 1.45 mL/kg/min) showed a clinically meaningful increase in peak VO_2_ with the caveat that these subgroups only included three studies.

Besides, our study did not show a significant improvement in exercise capacity with longer duration of exercise regime and higher number of sessions. A similar unchanged level of peak VO_2_ with a longer duration was seen in the Mueller study, where the peak VO_2_ did not show any significant interactions between both intensities after 12 months. A possible explanation would be the low patient adherence, as, for example, ∼60% of patients completed 12 months of exercise in the OptimEx-Clin study, compared with 80% for 3 months.^9^ Research into strategies that improve exercise adherence suggests that provision of feedback and monitoring may be effective.^36^ Future trials, particularly those of longer durations, may adopt such strategies to improve patient adherence.

Nevertheless, compared with no exercise, 36 sessions of high-intensity exercise were observed to be associated with increased peak VO_2_ and each 1 mL/kg/min increase in peak VO_2_ conferred a 58% improvement in 5-year mortality.^37^ Exercise in HFpEF is also associated with improvements in other markers of exercise capacity such as metabolic equivalents of task (MET) scores in treadmill stress tests.^38^ HFpEF patients with higher MET scores and thus cardiopulmonary fitness had higher survival in a retrospective cohort study.^39^ Although suggested by observation studies, the clinical benefit of improved peak VO_2_ associated with exercise training requires further validation in RCTs.

Exercise training also improves the quality of life of patients with HFpEF.^31,40^ The Exercise training in Diastolic Heart Failure Pilot (Ex-DHF-P) RCT showed that endurance/resistance training was associated with improved physical functioning, bodily pain, general health, social functioning, and mental component score on the 36-item Short-form Health Survey (SF-36).^40^ Exercise training was also associated with reduced symptoms of depression on the Patient Health Questionnaire (PHQ-9).^40^ Interestingly, quality of life is shown to be inversely associated with obesity and worse physical capacity and activity levels, including peak VO_2_ and 6 min walking distance, but not echocardiographic markers of diastolic function.^41^ Similarly, we found that exercise training improved quality of life on MLWHF questionnaire, but not markers of diastolic function. This has been shown in previous studies.^23,26^  *E*/*e*′ is the most established marker of diastolic dysfunction, but existing data only show a modest correlation with invasive filling pressures and outcomes in HFpEF.^42^ This suggests that quality of life and exercise capacity are important endpoints of clinical significance, and exercise training has positive impacts on both, while the role of echocardiographic diastolic markers as exercise determinants requires further investigation.

Exercise training improves exercise capacity and quality of life, yet, how much exercise causes improvements is currently uncertain. There have been contradicting results for the effects of high vs. moderate exercise in exercise capacity in HFpEF.^9,12,22^ The previous meta-analysis by Boulmpou *et al*.^32^ included two studies on 34 patients that compared HIIT with aerobic exercise, and reported a significantly greater improvement in peak VO_2_ with HIIT. In contrast, the recently published OptimEx-Clin RCT involving 176 patients randomized to HIIT, MCT, and guideline control showed no significant differences in peak VO_2_ between HIIT and MCT.^9^ With the addition of this study by Mueller *et al*.^9^ in our meta-analysis, we found that HIIT was associated with a significant but not clinical meaningful improvement in exercise capacity when compared with MCT. In addition, these studies have highlighted the concerns regarding adherence of the participants to the training regime,^9,12,22^ which perhaps points to the difficulty in translating training as a therapeutic target in clinical settings.

### Limitations

This meta-analysis has several limitations. First, many of the RCTs included were performed on very small trial populations, and the studies were unblinded due to the nature of the intervention. It is likely due to many challenges associated with exercise training highlighted in previous studies, for example, the difficulty with adherence and limitation of patient recruitment to those able to exercise.^9,31^ Secondly, the follow-up durations in these studies differed, and the mean follow-up duration was relatively short. Thirdly, the definition of HFpEF, including EF cut-off, used in the studies was different, perhaps due to the challenge of diagnosing HFpEF and evolving diagnostic criteria recommended by international guidelines.^43^ This is reflected in the varied baseline characteristics in the participants among the included studies. Additionally, there are no exercise data reported across baseline characteristics such as age which limits the plausibility of the results. Lastly, the result of the funnel plot for the primary outcome suggests the presence of publication bias. Considering that all the included studies, except one, had fewer than 50 subjects in each arm, studies in this field may be susceptible to small-study effects. Future studies should aim to recruit a greater number of subjects to reduce the likelihood of a small-study effect.

## Conclusions

Exercise increases the exercise capacity and quality of life in HFpEF patients, and high-intensity exercise is associated with a small but significant improvement in exercise capacity than moderate intensity. There is, however, no significant improvement with longer duration and higher number of sessions. Studies with a larger participant population with longer follow-up are warranted to determine the long-term effects of high- and moderate-intensity exercise.

Exercise increases the exercise capacity and quality of life in HFpEF patients, and high-intensity exercise is associated with small but statistically significant improvement in exercise capacity than moderate intensity. The clinical significance of this observed difference is questionable and needs further attention. Additionally, there is no significant improvement with longer duration and higher number of sessions. Studies with a larger participant population with longer follow-up are warranted to determine the long-term effects of high- and moderate-intensity exercise.

## Lead author biography


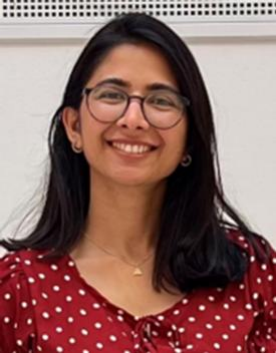


Ranu Baral, MBBS, MRes, graduated from Norwich Medical School in the UK with a degree in medicine and an intercalated Master of Research degree in 2019. She completed her initial training in Norwich and subsequently commenced internal medical training in South-East London. Dr Baral has a longstanding interest in heart failure and her primary research focuses on novel therapeutics in heart failure with preserved ejection fraction.

## Supplementary Material

oeae033_Supplementary_Data

## Data Availability

The data underlying this article will be shared on reasonable request to the corresponding author.
