# Supplementary material for: Exercise training improves exercise capacity and quality of life in heart failure with preserved ejection fraction: a systematic review and meta-analysis of randomized controlled trials
Source: Eur Heart J Open. 2024 Jun 26;4(4):oeae033. doi: 10.1093/ehjopen/oeae033 (PMC11231943; doi:10.1093/ehjopen/oeae033)

**Supplemental table 1:** Risk of Bias Assessment in the included trials.

| Study name | Selection bias | | Performance bias | Detection bias | Attribution bias | Reporting bias |  |
| --- | --- | --- | --- | --- | --- | --- | --- |
|  | Random sequence generation | Allocation concealment | Blinding of participants and personnel | Blinding of outcome assessment | Incomplete outcome data | Selective reporting | Other bias |
| Gary 2004 | Low | Unclear | High | Unclear | Unclear | Low | Unclear |
| Kitzman 2010 | Low | Unclear | Low | Low | Low | Low | Unclear |
| Edelmann 2011 | Low | Unclear | Unclear | Low | Low | Low | Unclear |
| Alves 2012 | Unclear | Low | High | Low | Low | Low | Unclear |
| Smart 2012 | High | Unclear | Unclear | Low | Low | Low | Unclear |
| Karavidas 2013 | Low | Low | Unclear | Low | Low | Unclear | Unclear |
| Kitzman 2013 | Low | Unclear | Low | Low | Low | Low | Unclear |
| Palau 2014 | Unclear | Unclear | Unclear | Low | Low | Low | Unclear |
| Angadi 2015 | Unclear | Unclear | Unclear | Low | Low | Low | Unclear |
| Kitzman 2016 | Low | Low | Low | Low | Low | Low | Unclear |
| Palau 2019 | Unclear | Unclear | Unclear | Low | Low | Low | Unclear |
| Kinugasa 2020 | Unclear | Unclear | High | High | Unclear | Unclear | Unclear |
| Silveria 2020 | Low | Low | Unclear | Low | Low | Low | Unclear |
| Mueller 2021 | Low | Unclear | Low | Low | Low | Low | Unclear |

**Supplemental Figure 1**: Search strategy


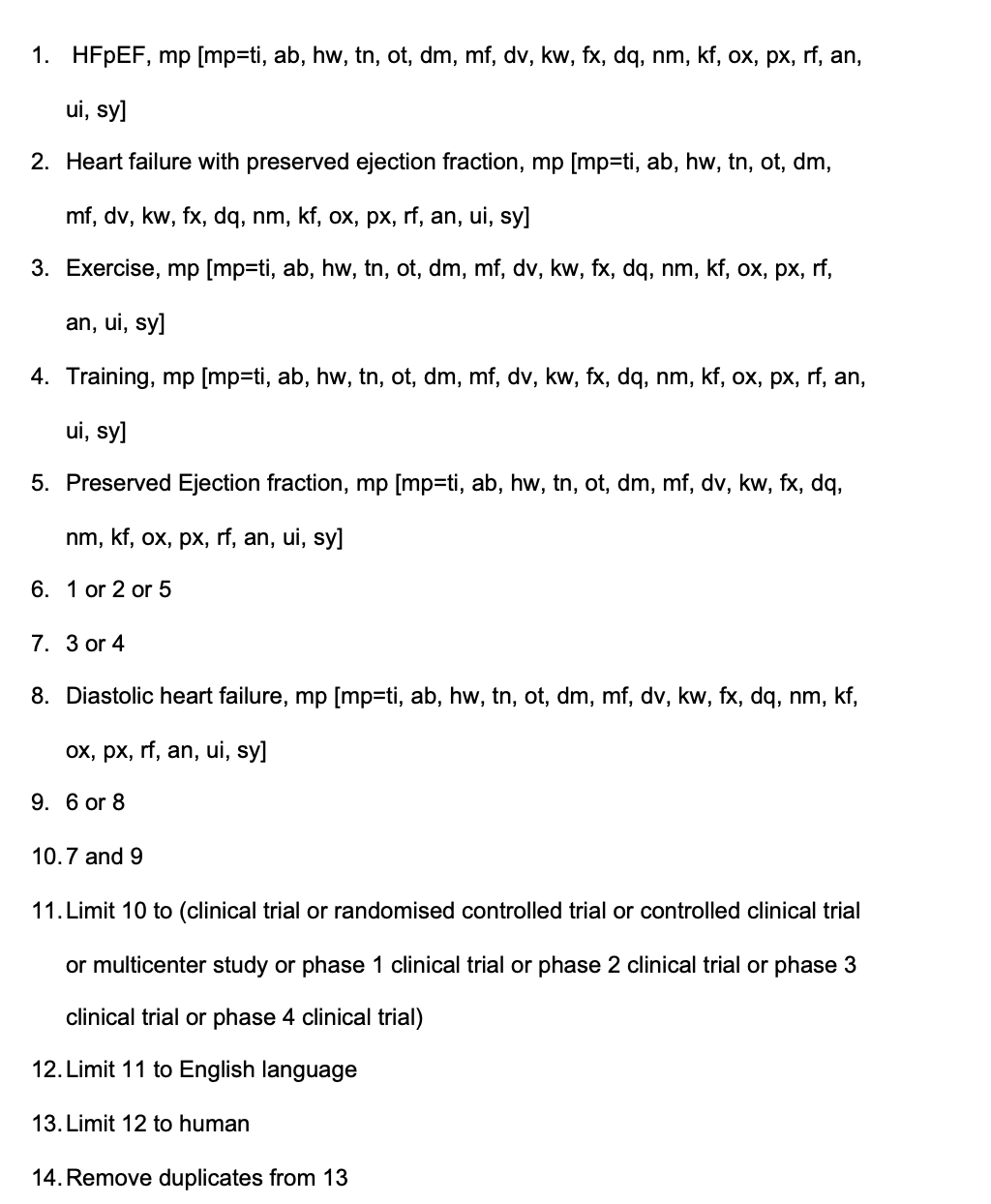


**Supplementary Figure 2:** Funnel plot of the studies reporting peak VO2


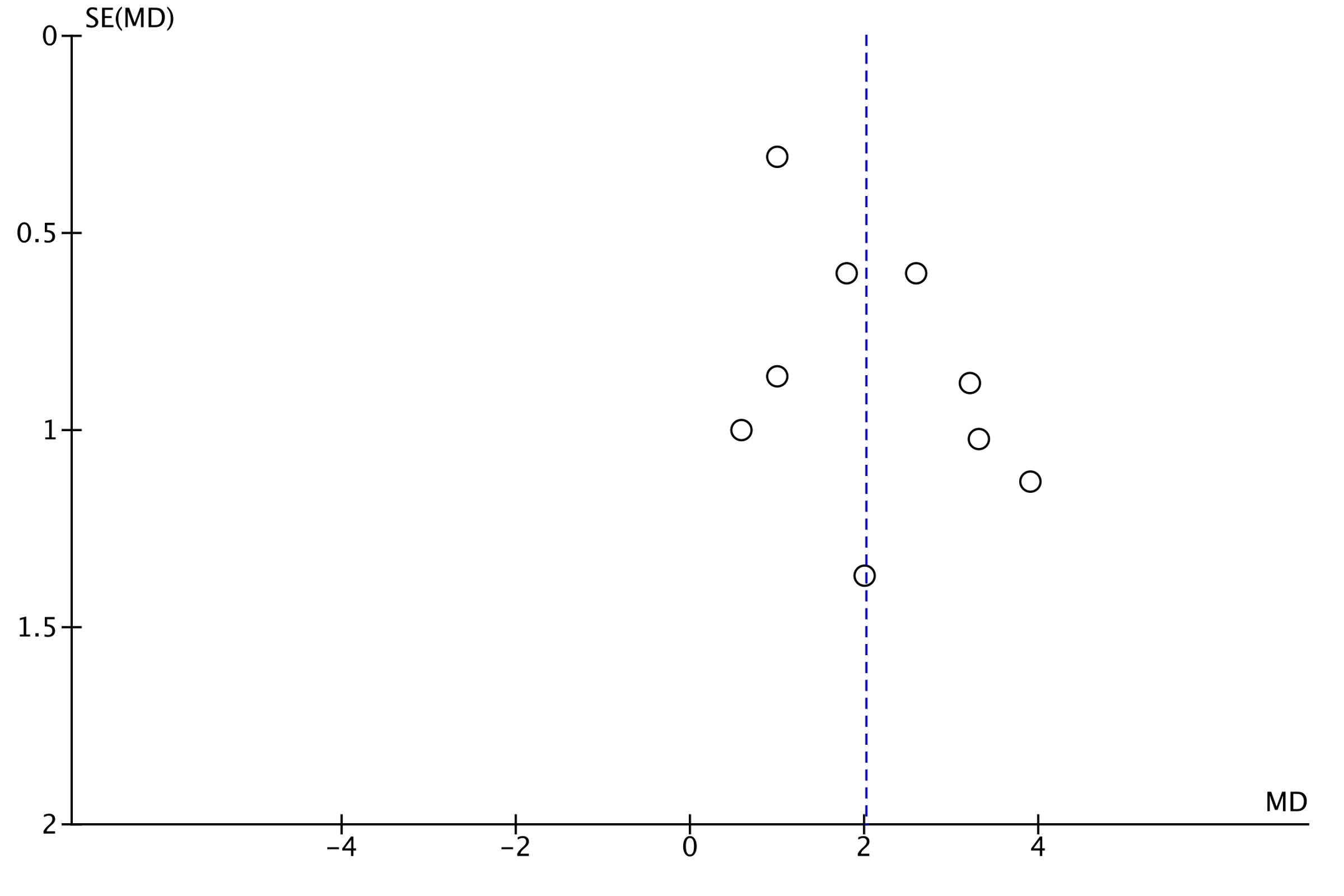


**Supplementary Figure 3:** Forest plot showing effect of exercise training on 6-minute walk test (6-MWT) among participants with heart failure and preserved ejection fraction. CI indicates confidence interval; and MD, mean difference.

**
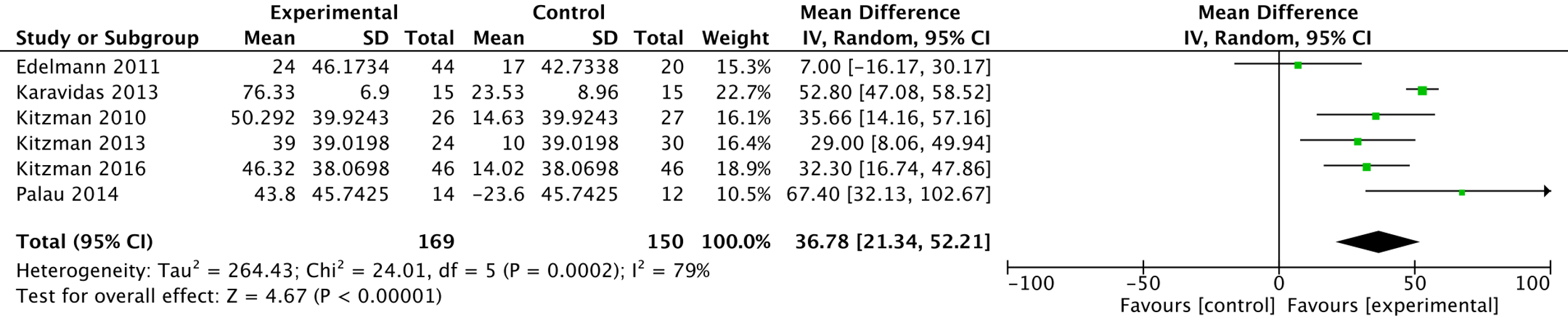
**


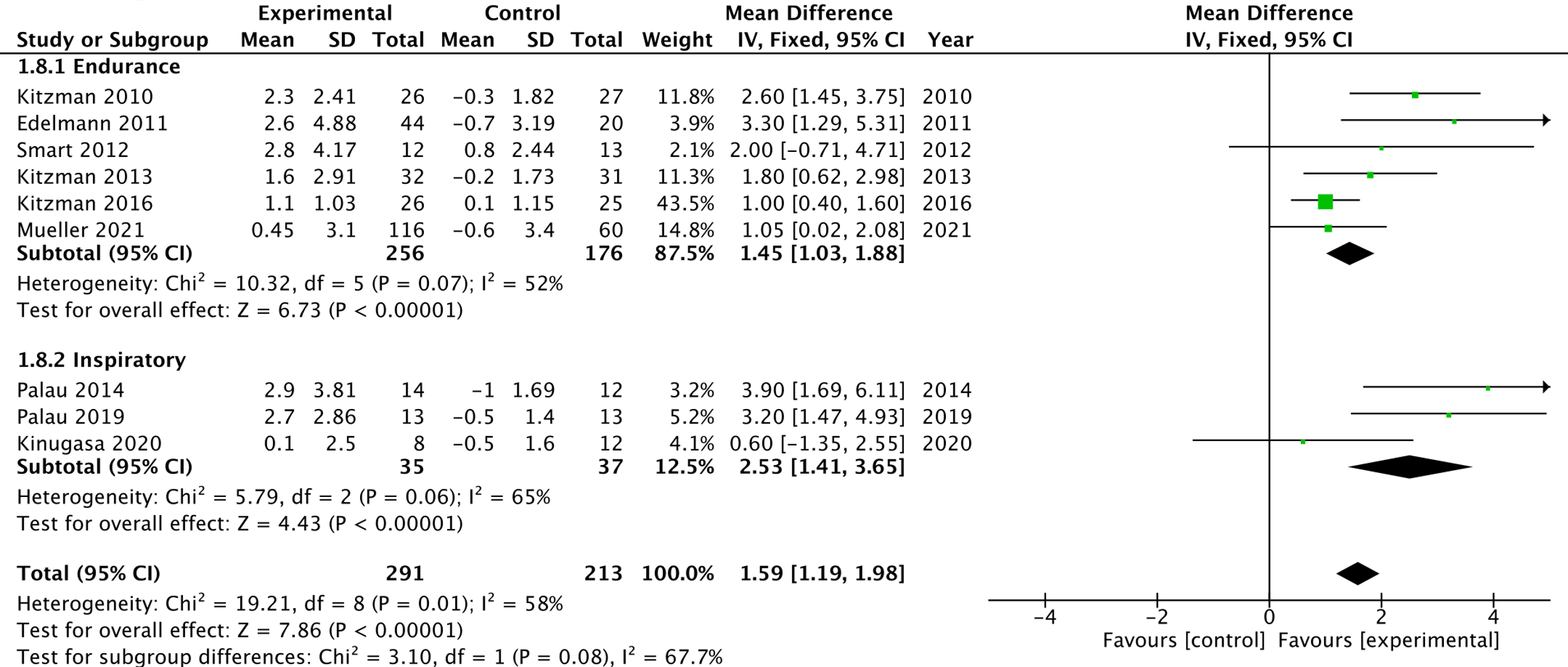
**Supplementary Figure 4:** Forest plot showing effect of exercise training (endurance vs inspiratory) on peak oxygen uptake (VO_2_) among participants with heart failure and preserved ejection fraction. CI indicates confidence interval; and MD, mean difference.

**Supplementary Figure 5:** Forest plot showing effect of exercise training of duration <=12 vs >= 16 weeks on peak oxygen uptake (VO_2_) among participants with heart failure and preserved ejection fraction. CI indicates confidence interval; and MD, mean difference.


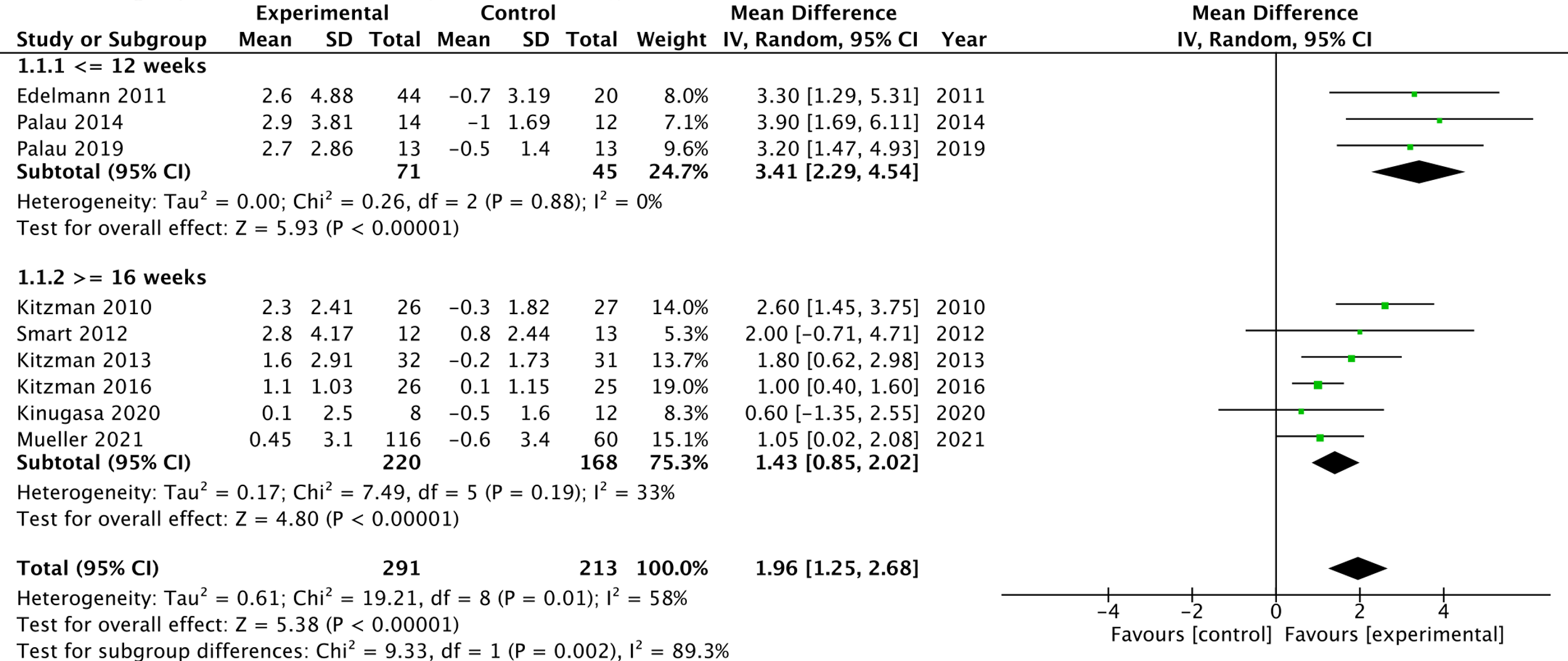


**Supplementary Figure 6:** Forest plot showing effect of exercise training on number of <=48 vs >=60 sessions on peak oxygen uptake (VO_2_) among participants with heart failure and preserved ejection fraction. CI indicates confidence interval; and MD, mean difference.

**
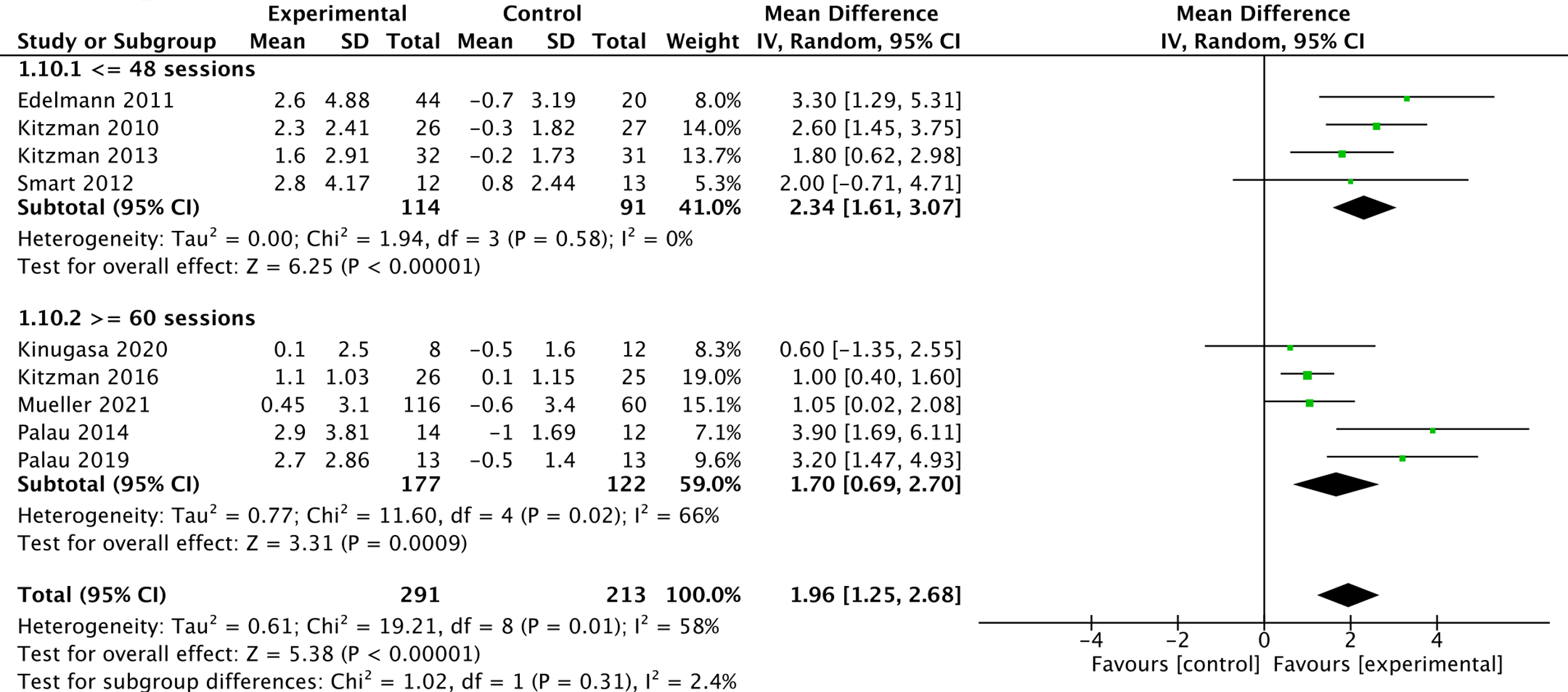
**

**Supplementary Figure 7:** Meta-regression of mean difference in peak VO_2_ and duration of exercise training (A) and number of exercise sessions (B).

A


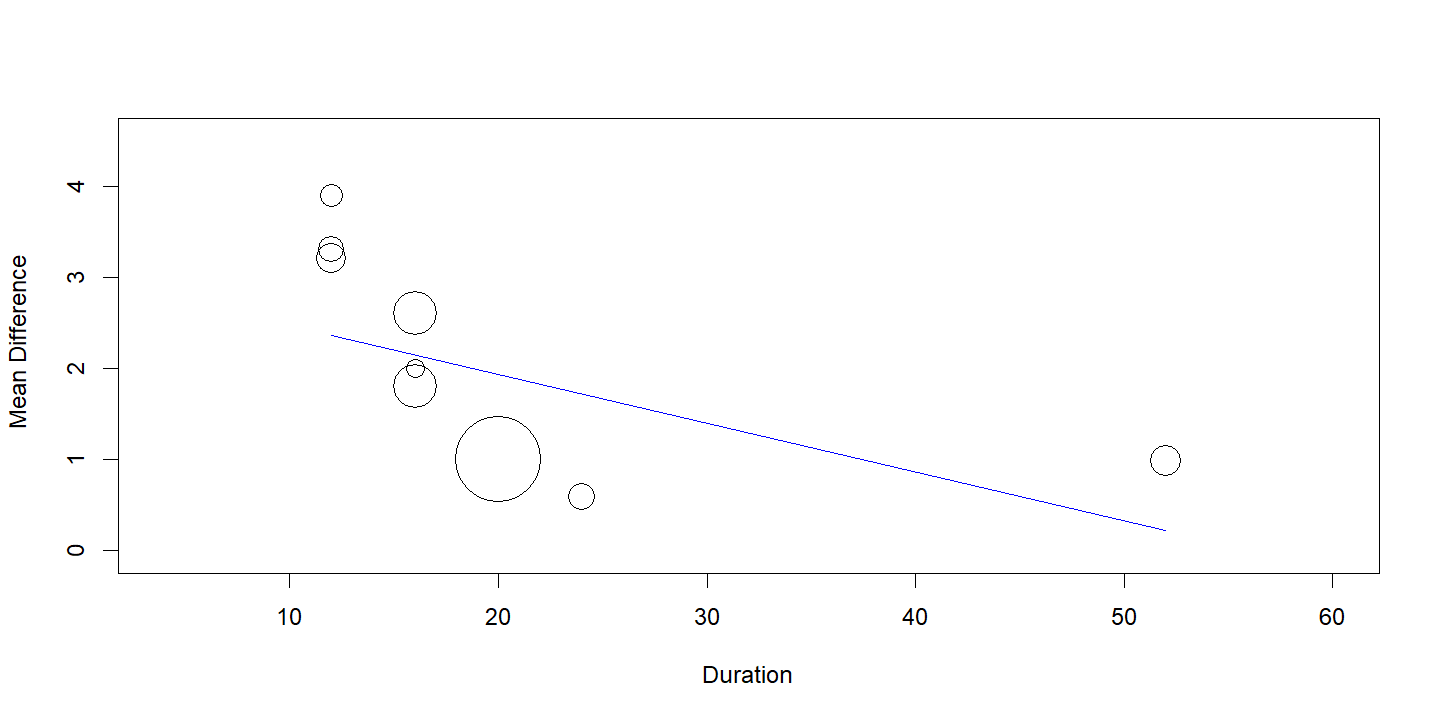


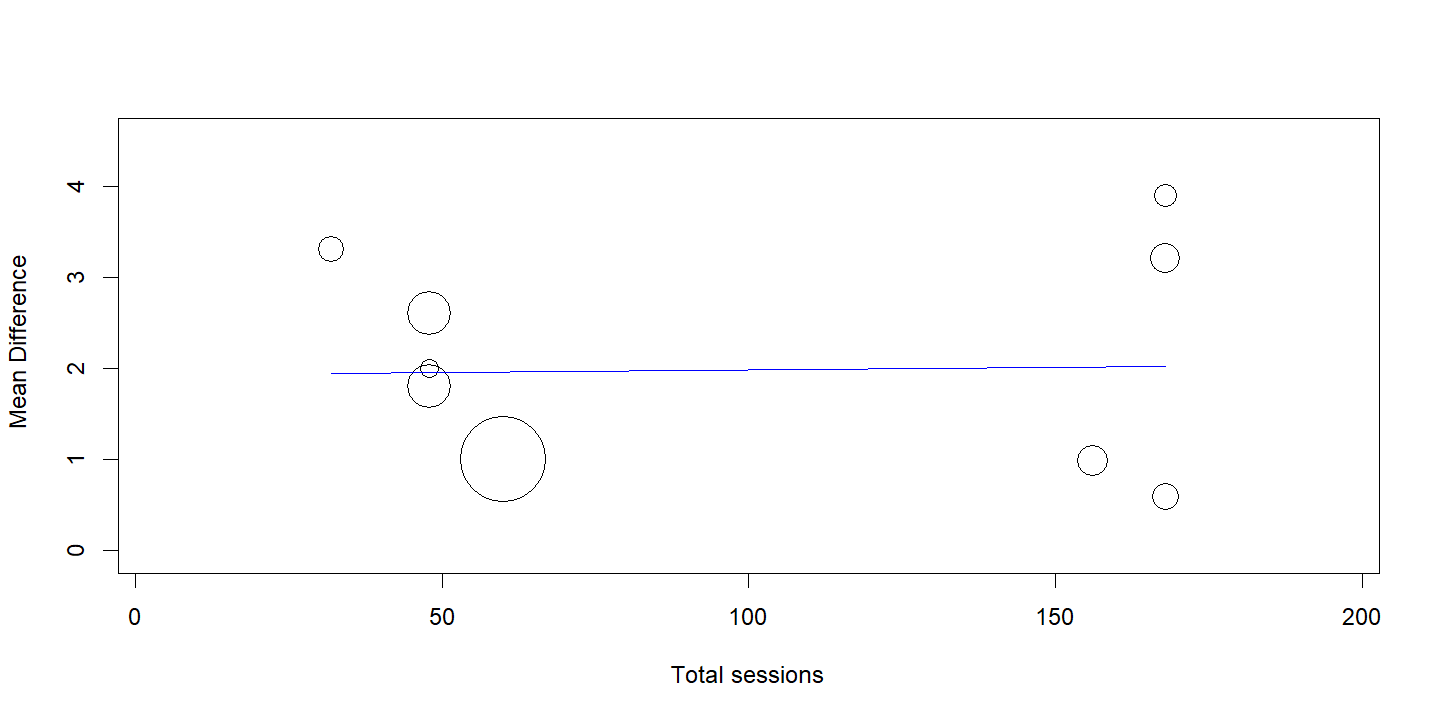
B

**Supplementary Figure 8:** Sensitivity analysis showing effect of exercise training on MLWHF questionnaire excluding Karavidas study


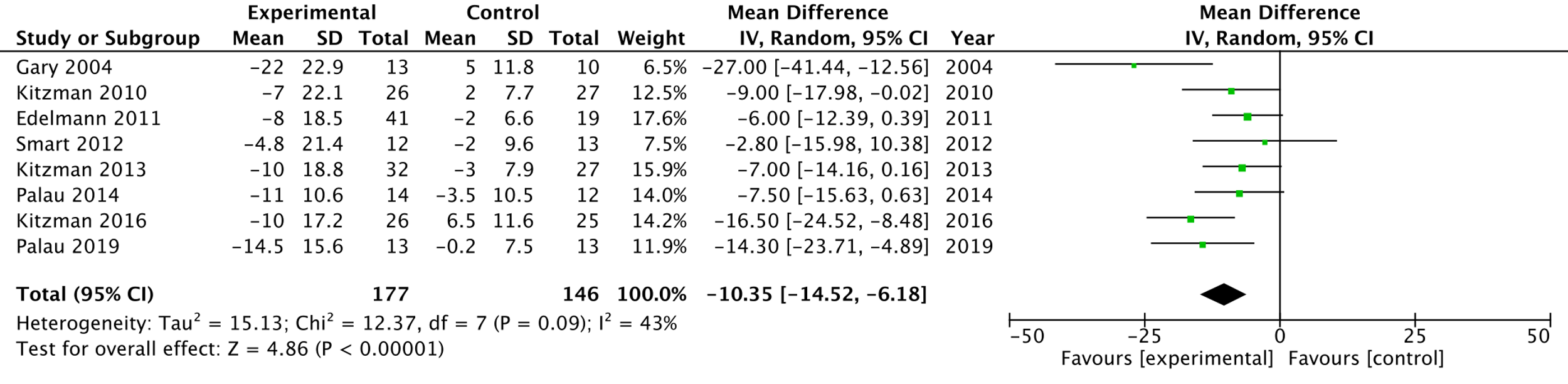

Supplement: oeae033_Supplementary_Data [file oeae033_supplementary_data.docx]
